# Urea-Doped ZnO Films as the Electron Transport Layer for High Efficiency Inverted Polymer Solar Cells

**DOI:** 10.3389/fchem.2018.00398

**Published:** 2018-09-07

**Authors:** Zongtao Wang, Zhongqiang Wang, Ruqin Zhang, Kunpeng Guo, Yuezhen Wu, Hua Wang, Yuying Hao, Guo Chen

**Affiliations:** ^1^Key Laboratory of Interface Science and Engineering in Advanced Materials, Ministry of Education, Research Center of Advanced Materials Science and Technology, Taiyuan University of Technology, Taiyuan, China; ^2^Key Laboratory of Advanced Display and System Applications, Ministry of Education, Shanghai University, Shanghai, China

**Keywords:** polymer solar cells, ETL, ZnO, urea, PCE

## Abstract

In this paper, urea-doped ZnO (U-ZnO) is investigated as a modified electron transport layer (ETL) in inverted polymer solar cells (PSCs). Using a blend of Poly{4,8-bis[(2-ethylhexyl)oxy] benzo [1,2-b:4,5-b'] dithiophene-2,6-diyl-alt-3-fluoro-2-[(2-ethylhexyl)carbonyl] thieno [3,4-b] thiophene-4,6-diyl}(PTB7), and [6,6]-phenyl-C71-butyric acid methyl ester (PC_71_BM) as light absorber, a champion power conversion efficiency (PCE) of 9.15% for U-ZnO ETL based PSCs was obtained, which is 15% higher than that of the pure ZnO ETL based PSCs (7.76%). It was demonstrated that urea helps to passivate defects in ZnO ETL, resulting in enhanced exciton dissociation, suppressed charge recombination and efficient charge extraction efficiency. This work suggests that the utilization of the U-ZnO ETL offer promising potential for achieving highly efficient PSCs.

## Introduction

In past decades, the need of green and sustainable energy has become more and more pressing. Polymer solar cells (PSCs) as one of the most potential renewable energy technologies have attracted wide attention due to its merits, such as low cost, light weight, and the capability of fabricating flexible large-area modules (Zhang et al., 2016; Sun et al., 2017; Tran et al., 2017; Hou et al., 2018; Li et al., 2018). To improve the performance of PSCs, enormous efforts have been made by the worldwide researchers. Benefited from the progresses of new optoelectronic materials and interfacial engineering, the power conversion efficiency (PCE) have reached over 14% in single-junction PSCs (Gao et al., 2016; Liu et al., 2016; Wu et al., 2016; Li et al., 2017; Peng et al., 2017). Meantime, it was found the interfacial engineering plays key role in determining the efficiency and the stability of PSCs (Liu, Z. et al., 2017). For example, it has been demonstrated that the application of anode and electrode interlayer leads to 20% improvement compared to the reference device without electrode interlayer (Wu et al., 2016). The interfacial contact at the interface between active layer and electrode usually polished by inorganic and organic materials (such as graphene, carbon quantum dots, PEIE, PEI), which benefits the charge transport and extraction in PSCs, resulting in enhanced device performance (Bi et al., 2018).

In conventional structure devices, low work-function metal and air sensitive materials are widely used as electron extraction layer (Liu, D. et al., 2017; Huai et al., 2018), which limits the stability of devices. Hence, the inverted device structure is developed in PSCs, which switches the position of anode and cathode, preventing the interfacial deterioration and physical degradation of organic layers (Wang et al., 2012; Cheng et al., 2018). Therefore, the device stability can be significantly improved in inverted structure devices. In addition, the inverted PSCs showed excellent performance due to the efficient charge extraction and suppressed recombination loss compared to the conventional structure devices (Wang et al., 2017).

In inverted PSCs, the metal-oxide materials have been utilized as buffer layers to extract and transport charge carriers, such as TiO_x_, CsO_x_, SnO_2_, ZnO (Tozlu et al., 2017; Tran et al., 2017; Wang et al., 2017; Jung et al., 2018). Due to the excellent electron extraction property, high electron mobility and easy processing, environment-friendly ZnO is widely used in the inverted PSCs (Vohra et al., 2015). However, Sol-Gel processed ZnO films exhibit certain defects on the surface and inside, which limit the application as electron transport layer (ETL) in the highly efficient PSCs (Gu et al., 2014). These defects are apt to cause electron trap loss in PSCs, resulting in the severe charge carrier recombination loss. Additionally, poor contact between inorganic and organic materials also cause bad electrical contact, leading to recombination loss (Han et al., 2016).

To facilitate the electron extraction in PSCs, many attempts have been made to polish the interface between ZnO ETL and active layer (Zhang et al., 2018). The double-layer structure of ZnO/organic was developed to modify ZnO ETL and prevent direct contact of ZnO ETL with active layer (Zhang et al., 2018). Compared to pristine ZnO ETL, double-layer structure ZnO/organic ETL showed impressive enhancement in PCE. Following this principle, various materials, such as ionic liquids, polyelectrolytes, self-assembled monolayers, have been introduced to form double-layer structure ETL in PSCs (Dai et al., 2011; Zhang et al., 2018). However, new interfaces are introduced into the devices after the application of double-layer structure ETL, resulting in new interfacial contact. Thus, it is highly desirable but challenged to develop efficient and simple ETL in PSCs.

In order to address the above mentioned problems, we envisaging that modifying ZnO with suitable material would help to eliminate or passivate defects in ZnO films. To realize this, we noticed urea would be a good dopant candidate. This is because the possibility of formation of coordinate bonds between the lone pair of electrons on the two nitrogen atom of the amino groups and the Lewis acid sites of the ZnO surface would enable defects reducing.

In this work, urea-doped ZnO (U-ZnO) was investigated as an electron transport layer (ETL) in inverted PSCs with a blend of Poly{4,8-bis[(2-ethylhexyl)oxy]benzo[1,2-b:4,5-b']dithiophene-2,6-diyl-alt-3-fluoro-2-[(2-ethylhexyl)carbonyl]thieno[3,4-b]thiophene-4,6-diyl} (PTB7), and [6,6]-phenyl-C71-butyric acid methyl ester (PC_71_BM) as active layer. With the device structure of ITO/ U-ZnO (40 nm)/PTB7:PC_71_BM(95 nm)/MoO_3_(5 nm)/Al (80 nm), an optimal PCE value of 9.15% was achieved, which is 15% higher than that of the pure ZnO ETL based reference device (7.76%). The performance enhancement of the U-ZnO ETL based PSCs may be attributed to the promotion of exciton dissociation, the suppression of charge recombination and the improvement of charge extraction capability. This investigation proves that the U-ZnO ETL offers promising potential for achieving high efficiency in PSCs.

## Experimental

### General information

PTB7, PC_71_BM, and MoO_3_ were purchased from 1-Material Company of United States, Luminescence Technology Corporation of Taiwan and Rieke Company of China, respectively. Anhydrous zinc acetate [Zn(CH_3_COO)_2_] and 2-methoxyethanol (CH_3_OCH_2_CH_2_OH) were obtained from Energy Chemical. Ethanolamine (NH_2_CH_2_CH_2_OH) was purchased from Sigma-Aldrich Company. All materials and solvents were used as purchased without further purification.

### Device fabrication

To obtain the ZnO precursor solution, 0.836 g of Zn(CH_3_COO)_2_ and 0.28 g of NH_2_CH_2_CH_2_OH were dissolved in 10 mL of CH_3_OCH_2_CH_2_OH. Different ratios of urea were added into CH_3_OCH_2_CH_2_OH to prepare the U-ZnO precursor. All the precursor solutions were stirred overnight in air. A blend of PTB7:PC_71_BM (1:1.5 *wt* %, 25 mg mL^−1^) was dissolved in the mixed solvents of CB and DIO (97:3 *vol*%) and was stirred overnight at 50°C. Pre-patterned ITO substrates (15 Ω/square) were cleaned in detergent, acetone, and isopropanol for 30 min in sequence. Then the substrates were dried overnight in an oven with a temperature of 50°C. The ultraviolet-ozone treated ITO substrates were used to spin coat ZnO precursor or U-ZnO precursor at 4000 rpm in air. Then the ZnO-coated or ZnO-coated substrates were annealed at 200°C for 1 h in air. After that, the substrates were put into a N_2_-filled glovebox for active layer spin-coating. Then the samples were quickly transfer into vacuum chamber for electrode deposition. Finally, 5 nm MoO_3_ and 80 nm Al were deposited in a vacuum chamber with base press of 1 × 10^−4^ Pa.

### Characterization

The current density-voltage (*J-V*) characteristics, PCE and external efficiency quantum (EQE) were recorded by a Newport solar simulator system. The *J-V* characteristics were recorded under AM 1.5G illumination with light intensity of 100 mW cm^−2^. A calibrated silicon diode was set as the reference, which exhibited a response from 300 to 800 nm. The photoluminescence (PL) samples were measured by A Hitachi F-7000 spectrofluorophotometer.

## Results and discussion

**Figure 1:**
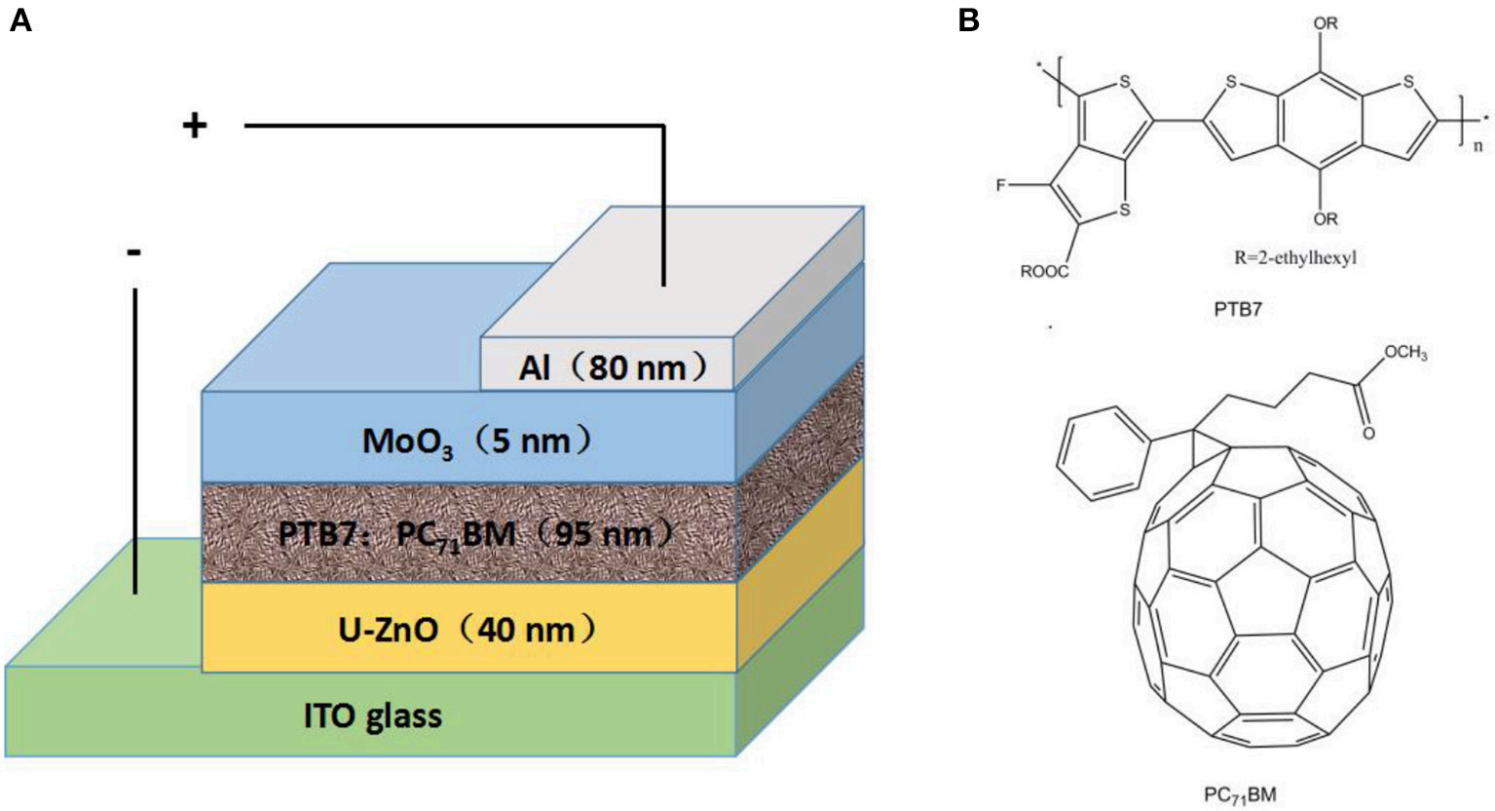
**(A)** Device architecture of inverted polymer solar cells. **(B)** Molecular structures of PTB7 and PC_71_BM.

The device architecture of inverted PSCs was shown in Figure 1A, with a structure of ITO/U-ZnO(40 nm)/PTB7:PC_71_BM(95 nm)/MoO_3_(5 nm)/Al. According to our previous study, the thicknesses of ETL and active layer were set to the optimal values of 40 and 95 nm in the device fabrication, respectively(Zhang et al., 2018). The molecular structures of PTB7 and PC_71_BM were illustrated in Figure 1B.

**Figure 2:**
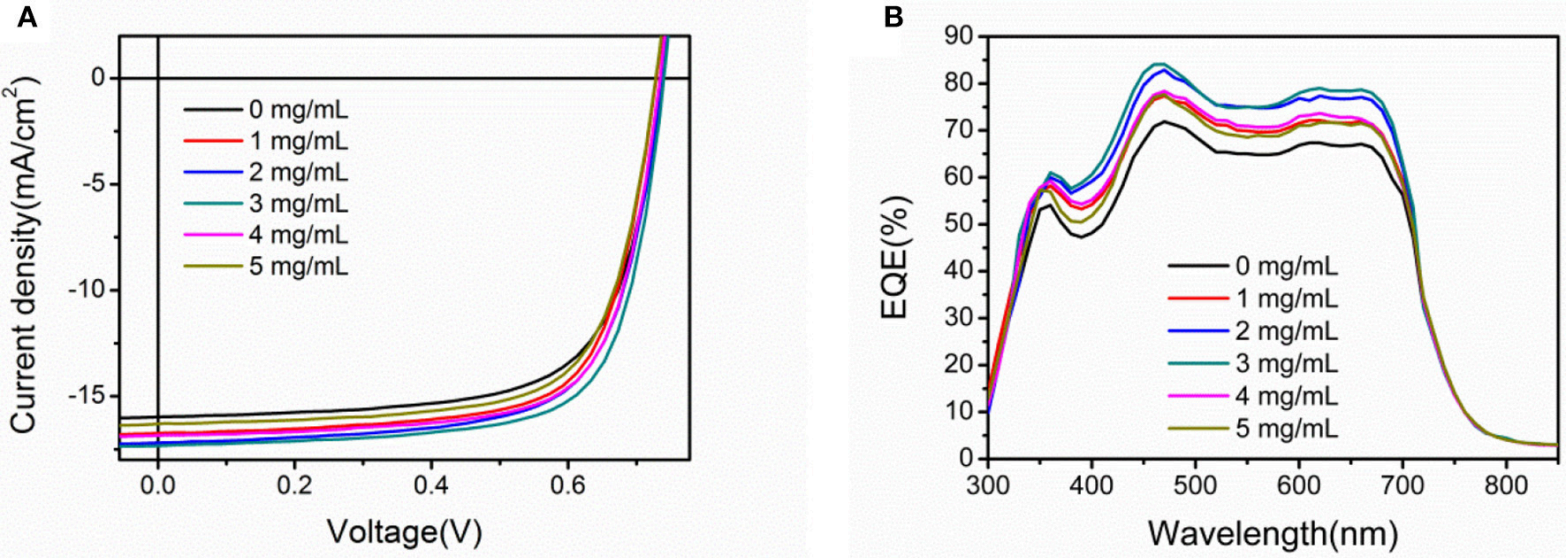
**(A)** Illuminated *J-V* characteristics and **(B)** EQE spectra in inverted PSCs using different concentrations U-ZnO as the ETLs. The structure of PSCs is ITO/ETL(40 nm)/PTB7:PC_71_BM(95 nm)/MoO_3_(5 nm)/Al(80 nm).

As a ETL in PSCs, high transmittance is the priority, which is highly related to the photovoltaic performance. As shown in Supporting Information Figure S1, the U-ZnO ETLs show high transmittance, which is slightly lower than pure ZnO ETL. Moreover, Supporting Information Figure S2 compares the light absorption in PTB7:PC_71_BM active layers. The light absorption spectra overlap each other, indicating good transmittance of U-ZnO ETL. To optimize the photovoltaic performance of PSCs, the urea doped concentration was tuned from 0 to 5 mg mL^−1^ in ZnO precursor solution. Figure 2A shows *J-V* characteristics and Figure 2B shows EQE photoresponse spectra of pure ZnO and U-ZnO ETLs based inverted PSCs. A summary of the corresponding photovoltaic parameters is listed in Table 1. The reference device exhibited an open circuit voltage(*V*_*oc*_) of 0.73 V, a short circuit current density(*J*_*sc*_)of 15.38 mA/cm^2^, a fill factor (FF) of 68.75%, and a PCE of 7.76%. In comparison with the reference device, the U-ZnO ETL based devices showed an obvious improvement in *J*_*sc*_ and FF, resulting in enhanced PCE. As a result of variations of *J*_*sc*_, FF and *V*_*oc*_ in inverted PSCs, a champion efficiency of 9.15% was obtained with a *V*_*oc*_ of 0.74 V, a *J*_*sc*_ of 17.31 mA/cm^2^, and a FF of 71.43% for the device using 3 mg mL^−1^ U-ZnO as the ETL. As listed in Table 1, the values of *J*_*sc*_, FF and PCE were increased by increasing the concentration of urea from 0 to 3 mg mL^−1^ and then decreased at high concentration > 3 mg mL^−1^. The increased *J*_*sc*_ was supported by the EQE profiles of the devices. The Supporting Information Figure S3 plots the current density (16.86 mA/cm2) of champion cell calculated from EQE, showing lower discrepancy with the *J*_*sc*_ (17.31 mA/cm^2^) extracted from *J-V* curve. The enhanced photovoltaic performance in PTB7:PC_71_BM based devices means the superior property of U-ZnO ETL in inverted PSCs.

**Table 1:** Photovoltaic parameters of inverted PSCs based on PTB7:PC_71_BM using U-ZnO as the ETL under AM 1.5G irradiation (100 m W cm ^−2^). The values of J_cal_ are calculated from the related EQE spectra.

**Concentration (mg mL^−1^)**	***V_*oc*_*** **(V)**	***J_*sc*_(J_*cal*_)*** **(mA/cm^2^)**	***FF*** **(%)**	***PCE*** **(%)**	**R_s_** **(Ω cm^2^)**	**R_sh_** **(Ω cm^2^)**
0	0.73 ± 0.01	15.38 ± 0.22(14.95)	68.75 ± 1.0	7.76 ± 0.20	41	5470
1	0.73 ± 0.01	16.74 ± 0.13(16.26)	70.40 ± 0.2	8.59 ± 0.11	36	9627
2	0.73 ± 0.01	17.18 ± 0.16(16.81)	71.08 ± 0.8	8.67 ± 0.22	32	9932
3	0.73 ± 0.01	17.31 ± 0.09(16.86)	71.43 ± 0.6	9.00 ± 0.15	27	9785
4	0.73 ± 0.01	16.85 ± 0.11(16.57)	70.81 ± 0.8	8.76 ± 0.14	33	9404
5	0.73 ± 0.01	16.32 ± 0.10(15.97)	69.86 ± 0.7	8.29 ± 0.06	36	9501

**Figure 3:**
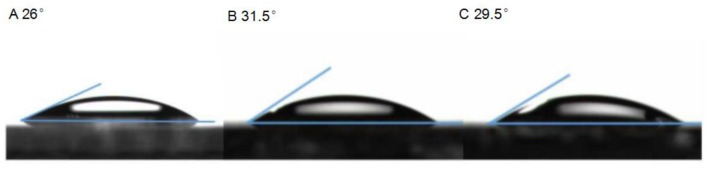
The water contact angle (θ) of different ratios U-ZnO ETLs, **(A)** 0 mg mL^−1^, **(B)** 3 mg mL^−1^, **(C)** 5 mg mL^−1^.

To investigate the interface contact between U-ZnO ETL and active layer, the contact angle of water droplets on pure ZnO and U-ZnO ETLs were measured in this study (Zisman, 2008; Peng et al., 2017; Han et al., 2018). As shown in Figure 3, the contact angles of water on 0, 3, 5 mg mL^−1^ U-ZnO ETLs were 26, 31.5 and 29.5°, respectively. In comparison with pure ZnO ETL, the U-ZnO ETL show much higher hydrophobicity, resulting in better interface contact between ETLs and photoactive layers. Thus, the better interface contact is beneficial to the improved performance of U-ZnO ETL based inverted PSCs.

**Figure 4:**
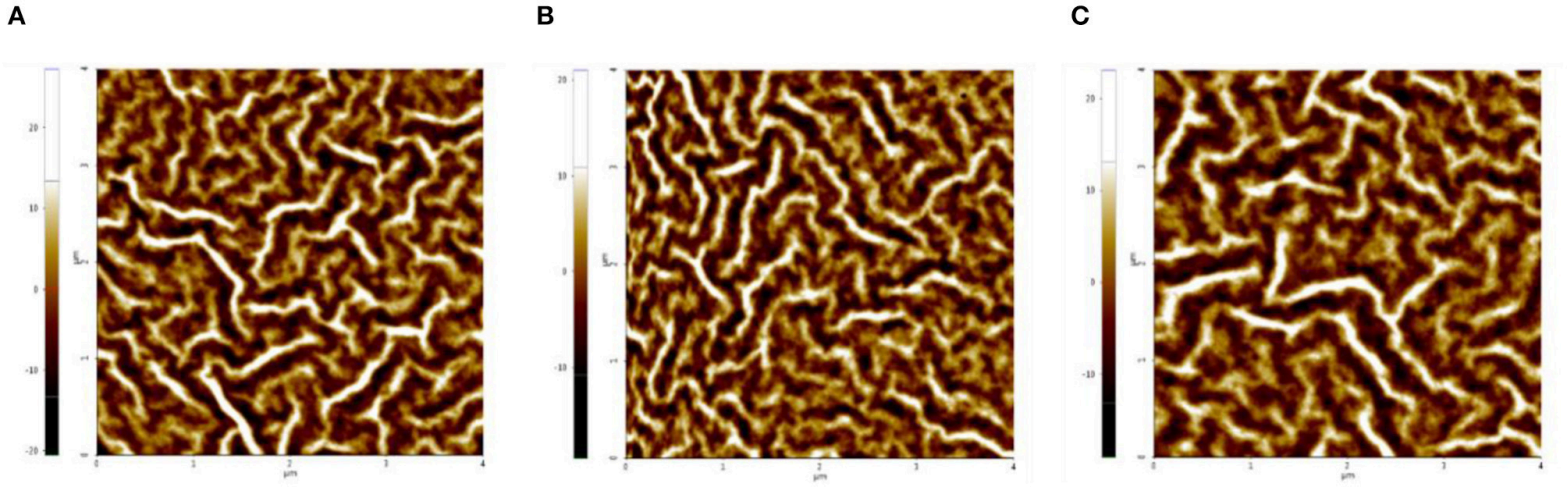
AFM top images (4.0 × 4.0 μm^2^) of the U-ZnO with different ratio of urea doping **(A)** 0 mg mL^−1^, **(B)** 3 mg mL^−1^, **(C)** 5 mg mL^−1^. The structure of samples is ITO/U-ZnO (40 nm).

It is known that the morphology of ETL is critical in determining the performance of PSCs(Tran et al., 2017). Hence, the surfacemorphology of the ETLs without and with the urea-doping are studied by atomic force microscopy (AFM), and the top images are displayed in Figure 4. The Root-Mean-Square (RMS) roughness of ZnO ETL without urea-doping is 1.19 nm. The RMS roughness is decreased to 0.74 and 1.14 nm in U-ZnO with 3 mg mL^−1^ and 5 mg mL^−1^, respectively. The smooth surface after urea-doping modifies the contact between ETLs and active layers, and infects the charge transport and collection in PSCs, leading to photocurrent improvement. On the other hand, the 2-D and 3-D images of PTB7:PC_71_BM active layers on U-ZnO ETLs are shown in Supporting Information Figure S4. AFM images of active layers reveal little morphology variation. The RMS surface roughness is slightly changed.

The space-charge-limited current (SCLC) method can be utilized to study the effect of urea on charge transport behavior inside the PSCs (Gupta et al., 2018). Hence, the electron mobility in ZnO and U-ZnO based devices were extracted by SCLC model. The electron-only device structures were ITO/ZnO or U-ZnO(40 nm)/PTB7:PC_71_BM(95 nm)/BCP(8 nm)/Al. As shown in Supporting Information Figure S5, the electron-only device *J-V* characteristics were fitted by SCLC model. The electron mobility in PSCs based on 0, 3, 5 mg mL^−1^ urea-doping U-ZnO ETLs are 1.87 × 10^−4^, 1.14 × 10^−3^, and 3.84 × 10^−4^ cm^2^ V^−1^ S^−1^, respectively. The superior electron mobility after urea-doping is beneficial to the electrical properties in PSCs, leading to efficient charge transport and extraction.

**Figure 5:**
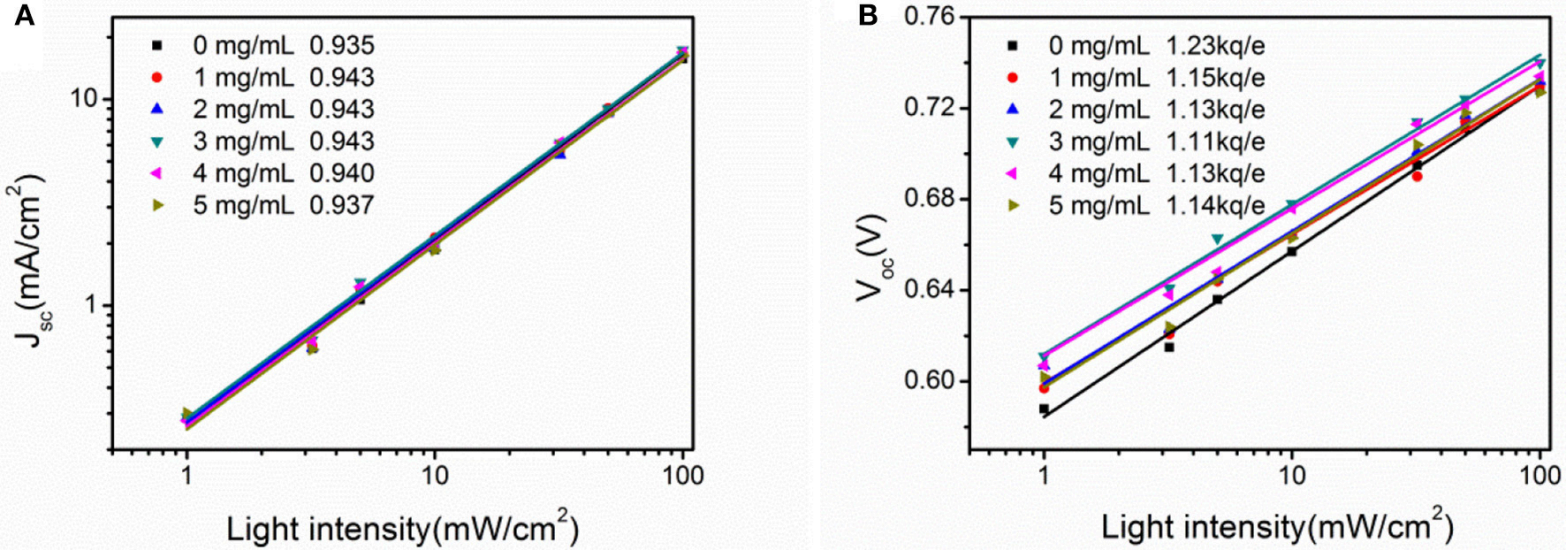
**(A)** *J*_*sc*_ of inverted PSCs as a function incident light intensity and **(B)** *V*_*oc*_ of inverted PSCs as a function of incident light intensity.

To understand the contribution of U-ZnO ETL in PSCs, the dependence of *J-V* characteristics with incident light intensity were compared in U-ZnO ETL based devices (Xiao et al., 2018). The plots in Figure 5A were fitted with power law, then the values of α were obtained (Schilinsky et al., 2002). As shown in Figure 5A, each α value is close to unity, indicating efficient charge transport and collection in these inverted PSCs at short circuit condition (Huang et al., 2018). Slightly improved α was observed after introduction of urea in precursor solution, which means more efficient charge transport and collection in inverted PSCs using U-ZnO as the ETL.

Furthermore, photo generated charge carriers recombine again inside of cells under the open circuit condition, which directly reflects the recombination loss of cells. Therefore, the dependence of *V*_*oc*_ with incident light intensity was studied in this work. The *V*_*oc*_ shows linear dependence with semi logarithmic incident light intensity with a slope of $\frac{KT}{e}$ (Yang et al., 2018), where *K* is the Boltzmann constant, *T* is the temperature in Kelvin, and *e* is the elementary charge. Figure 5B shows the linear dependence of *V*_*oc*_ as a function with incident light intensity and the extracted slope. It can be seen from the extracted slope that the values of slope are decreased after adding urea into ZnO precursor. Compared to the slope of $1.23\frac{KT}{e}\;$in PSCs using pure ZnO as the ETL, a smaller slope of $1.11\frac{KT}{e}$ is obtained when the concentration of urea in ZnO precursor solution is 3 mg mL^−1^, indicating the suppressed interfacial trap defects of the U-ZnO ETL. Consequently, the introduction of urea in ZnO precursor solution could passivate the defects of Sol-Gel processed ZnO ETL, resulting in improved *J*_*sc*_ and FF in U-ZnO ETL based PSCs.

**Figure 6:**
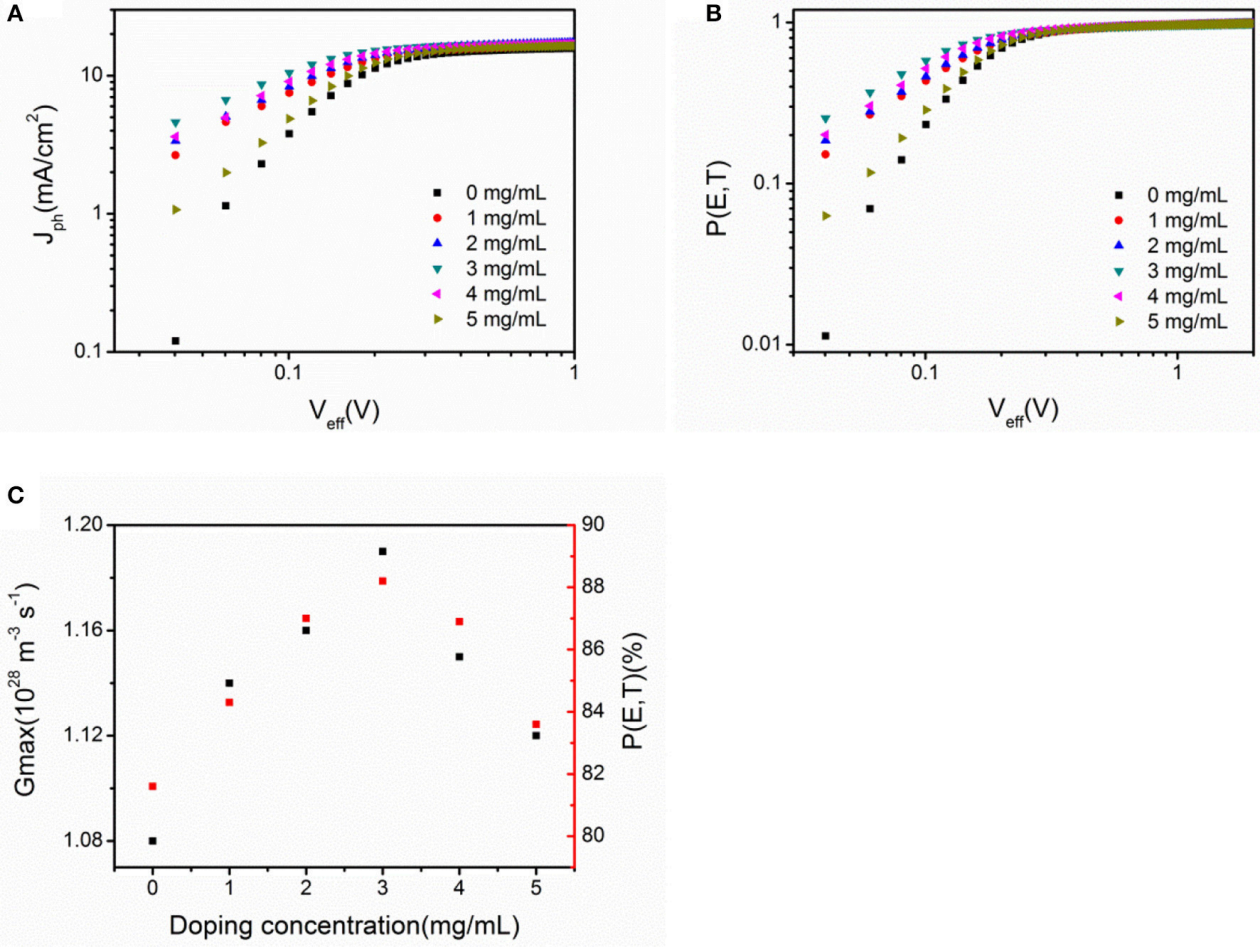
**(A)** Photocurrent density (*J*_*ph*_) as a function of the effective voltage (*V*_*eff*_). **(B)** Exciton dissociation probability *P (E, T*) as a function of the effective voltage. **(C)** The values of *G*_*max*_ and *P (E, T)* obtained for inverted PSCs using U-ZnO as the ETL. All PSCs have an identical configuration of ITO/ETL(40 nm)/PTB7:PC_71_BM(95 nm)/MoO_3_(5 nm)/Al(80 nm).

To further study the enhancement of *J*_*sc*_, the maximum exciton generation rate (*G*_*max*_) of PSCs was calculated in this study (Zhang et al., 2017). Figure 6A shows the dependence of photocurrent density (*J*_*ph*_) with the effective voltage (*V*_*eff*_). Apparently, two different regions were observed in *J*_*ph*_-*V*_*eff*_ characteristics. Under low effective voltage, the *J*_*ph*_ shows linear dependence with *V*_*eff*_. Then, it gradually approaches saturated value (*J*_*sat*_) under high effective voltage. The values of *G*_*max*_ are showed in Figure 6C. An obvious improvement in *G*_*max*_ for U-ZnO ETL based PSCs is clearly seen in Figure 6C, which means efficient charge carrier transport and extraction. The enhanced *G*_*max*_ highly contributes to the enhanced *J*_*sc*_ in U-ZnO ETL based PSCs.

In organic PSCs, only a portion of excitons dissociate into free charge carriers due to the unique optoelectronic conversion behavior. The exciton dissociation probability *P* (*E, T*) is related to electric field (*E*) and temperature (*T*). Hence, the value of *P* (*E, T*) under zero bias is deduced from Figure 6B. The value of *P (E, T)* increased from 81.6% in the control device to 88.2% in the U-ZnO ETL based device, implying that U-ZnO ETL can promote excitons dissociation. The improved excitons dissociation probability also contributes to the enhancement of *J*_*sc*_.

**Figure 7:**
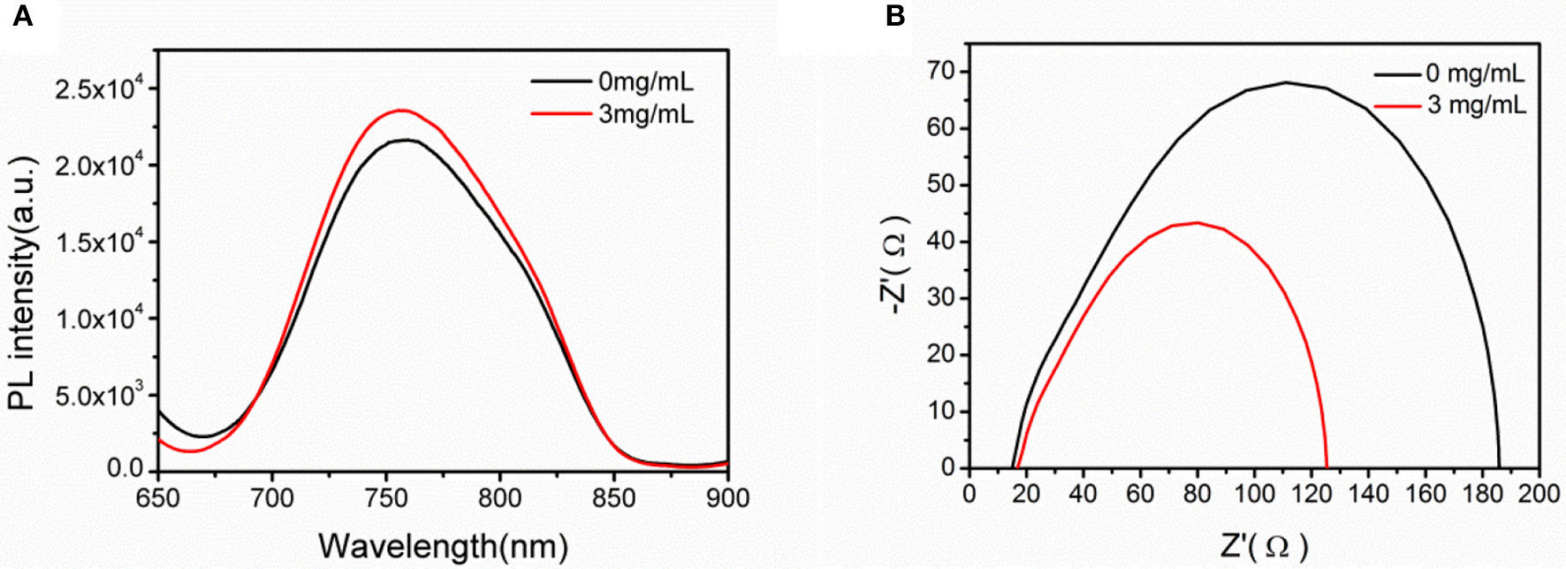
**(A)** PL spectra measured for device with a geometry of ITO/ETL(40 nm)/PTB7 (50 nm)/MoO_3_ (5 nm)/Al(10 nm) under photoexcitation of 600 nm at room temperature. **(B)** Impedance spectra (the Nyquist plot) of the devices with a structure of ITO/ETL(40 nm)/PTB7:PC_71_BM (95 nm)/MoO_3_ (5 nm)/Al (80 nm).

Due to the priority of exciton quenching in PSCs, photoluminescence (PL) spectra was used to analyze PL effect of pure ZnO and U-ZnO ETLs (Anger et al., 2006). Figure 7A shows the PL spectra of samples with a structure of ITO/ETL (40 nm)/PTB7 (50 nm)/MoO_3_ (5 nm)/Al (10 nm). In comparison with pure ZnO ETL, significant enhancement of PL intensity was observed in the U-ZnO ETL based samples, which indicated suppressed exciton quenching at the interface of ETL and active layer. The suppressed exciton quenching is beneficial to the improvement of *J*_*sc*_.

Electric impedance spectroscopy (EIS) is usually utilized to analyze the electrical behavior in PSCs (Zhang et al., 2018). Here, EIS spectrum is used to reveal the charge recombination inside of devices. The Nyquist plot of the impedance spectroscopy under dark condition was plotted in Figure 7B. Shorter diameter was observed after introduction of urea, meaning a lower transport resistance in U-ZnO ETL based devices. The decreased transport resistance reflects better contact between U-ZnO ETL and active layer, resulting in an efficient charge transport probability. The EIS spectra analyses also prove the efficient charge transport and extraction in U-ZnO ETL based PSCs.

## Conclusions

In summary, U-ZnO as the ETL was applied in PSCs. The advantages of U-ZnO ETL were analyzed in PTB7:PC_71_BM based inverted PSCs. The introduction of urea helps to passivate the defects of Sol-Gel processed ZnO ETL, polish the interface contact, and promote the exciton dissociation. In comparison with the devices using pure ZnO as the ETL, an impressive improvement was observed in PTB7:PC_71_BM based PSCs with the U-ZnO ETL. A champion efficiency of 9.15% was obtained with ~15% enhancement compared to the efficiency of 7.76% in pure ZnO ETL based PSCs. Our results suggest that U-ZnO ETL have great potential in organic PSCs.

## Author contributions

Device fabrication and photovoltaic performance studies were carried out by ZoW, RZ, and YW. ZhW, KG, HW, YH, and GC contributed to discussions. ZhW led the project, and prepared the manuscript. All authors contributed to the manuscript.

### Conflict of interest statement

The authors declare that the research was conducted in the absence of any commercial or financial relationships that could be construed as a potential conflict of interest.
