# Supplementary material for: Urea-Doped ZnO Films as the Electron Transport Layer for High Efficiency Inverted Polymer Solar Cells
Source: Front Chem. 2018 Sep 7;6:398. doi: 10.3389/fchem.2018.00398 (PMC6138008; doi:10.3389/fchem.2018.00398)
Supplement: Supplementary file 1 [file Table_1.docx]

**Supporting Information**

**Urea-doped ZnO Films as the Electron Transport Layer for High Efficiency Inverted Polymer Solar Cells**

Zongtao Wang^a^, Zhongqiang Wang^*a^, Ruqin Zhang^a^, Kunpeng Guo^*a^, Yuezhen Wu^a^, Hua Wang^a^ ,Yuying Hao^a^ and Guo Chen^b^

^a^Key Laboratory of Interface Science and Engineering in Advanced Materials, Ministry of Education, Research Center of Advanced Materials Science and Technology, Taiyuan University of Technology, Taiyuan, 030024 , China.

^b^Key Laboratory of Advanced Display and System Applications, Ministry of Education, Shanghai University, Yanchang Road 149, Shanghai, 200072, China

E-mail: [wangzhongqiang@tyut.edu.cn](mailto:wangzhongqiang@tyut.edu.cn); [guokunpeng@tyut.edu.cn](mailto:guokunpeng@tyut.edu.cn)

**

 FIGURE S1 │**Transmittance of U-ZnO ETL films with different ratio of urea doping 0 mg mL^-1^, 3 mg mL^-1^, 5 mg mL^-1^. The structure of samples is Glass/ITO/U-ZnO(40 nm).

**

**

**FIGURE S2 │** Light absorption comparison. The sample structure without urea-doping is Glass/ITO/ZnO(40 nm)/PTB7 (50 nm). The sample structure with urea-doping is Glass/ITO/U-ZnO(40 nm)/PTB7 (50 nm).





**FIGURE S3 │** The calculated current density (*J_sc_*) of device achieved from EQE spectrum with a champion efficiency of 9.15%. The structure of PSCs is ITO/U-ZnO(40 nm)/PTB7:PC_71_BM(95 nm)/MoO_3_(5 nm)/Al(80 nm). The doping concentration of urea is 3 mg mL^-1^.


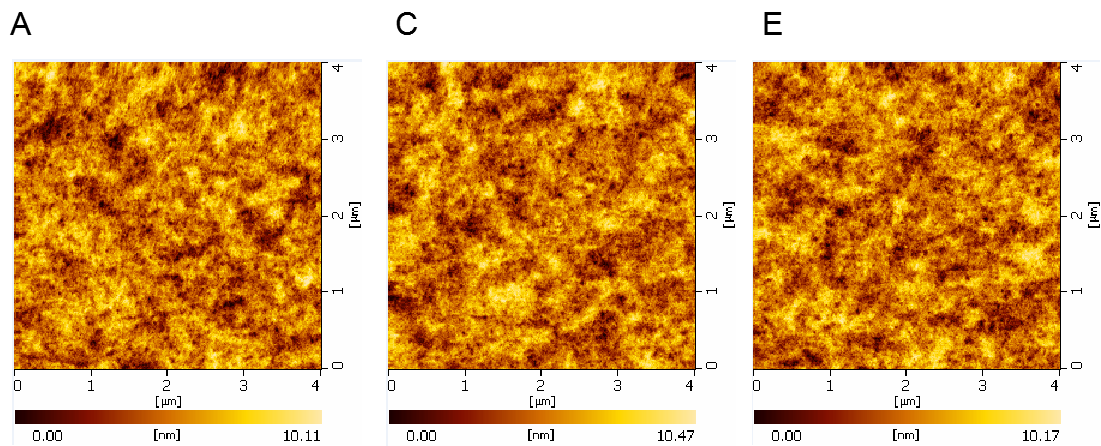


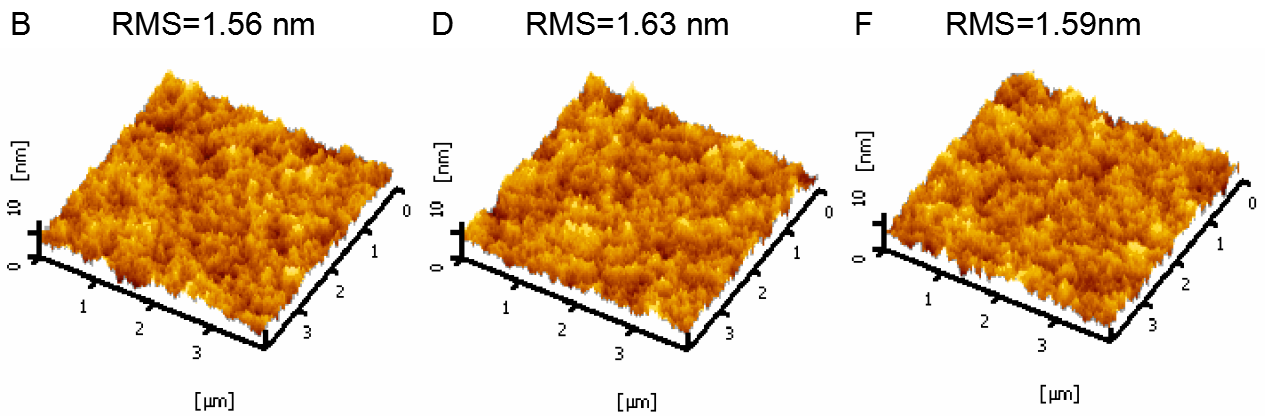


**FIGURE S4 │ (A)** 2-D and **(B)** 3-D AFM images (4.0×4.0 μm^2^) of active layer coated on pure-ZnO ETL. **(C)** 2-D and **(D)** 3-D AFM images (4.0×4.0 μm^2^) of active layer coated on U-ZnO(3 mg mL^-1^) ETL. **(E)** 2-D and **(F)** 3-D AFM images (4.0×4.0 μm^2^) of active layer coated on U-ZnO(5 mg mL^-1^) ETL. The sample structure with urea-doping is Glass/ITO/U-ZnO(40 nm)/PTB7:PC_71_BM (95 nm).





**FIGURE S5 │** *J-V* curves of the single-electron devices for ITO/ETL(40 nm)/PTB7:PC_71_BM(95 nm)/BCP(8 nm)/Al(80 nm).
